# Critical Assessment of Migration Strategies for Corrosion in Molten Salts

**DOI:** 10.3390/ma18163804

**Published:** 2025-08-13

**Authors:** M. Carmen Pavón-Moreno, Antonio Lopez-Paneque, Jose María Gallardo, Antonio Paul, Eduardo Díaz-Gutierrez, Cristina Prieto

**Affiliations:** 1 Department of Energy Engineering, Universidad de Sevilla, 41092 Sevilla, Spain; mpavonm@us.es; 2 Department of Materials, Universidad de Sevilla, 41092 Sevilla, Spain; alpaneque@us.es (A.L.-P.); josemar@us.es (J.M.G.); apaul@us.es (A.P.); eduardodiaz@us.es (E.D.-G.)

**Keywords:** corrosion, molten salts, materials, thermal energy storage

## Abstract

This review article examines the corrosion phenomena and mitigation strategies associated with molten salts used in thermal energy storage (TES) and heat transfer applications. Corrosion presents a critical challenge in concentrated solar power (CSP) plants and other high-temperature systems, affecting the durability and cost-efficiency of materials in storage tanks, heat exchangers, and piping. This study offers a comprehensive comparison of corrosion test methods and results, analyzing factors such as operating conditions, salt compositions, and material properties. Emphasis is also placed on strategies such as molten salt purification, the addition of corrosion inhibitors, and the application of protective coatings. This review aims to advance research and development in the TES sector by highlighting knowledge gaps and proposing directions for future experimentation.

## 1. Introduction

The global transition towards more sustainable production, with short-term goals such as decarbonization and the integration of renewable energy, has led to an increasing share of renewables in the energy market [1]. Since industrial energy consumption accounts for nearly 40% of total use [2], industrial decarbonization represents one of the most daunting challenges of the energy transition [3]. An evaluation of the energy consumption profile in this sector highlights the critical role played by process heat. One of the most promising current alternatives involves addressing both electricity and heat demands while maximizing the integration of renewable energy sources, particularly through system hybridization [4]. Most hybrid systems under consideration predominantly use existing concentrated solar power (CSP) plants, which benefit from technological maturity and integrated thermal energy storage (TES) systems [5,6]. These systems facilitate energy storage and offer a certain level of dispatchability. Additionally, other renewable generation technologies, such as photovoltaics and wind, are being incorporated due to their cost-effectiveness [7]. To further enhance the dispatchability of a hybrid system and reduce renewable energy curtailment during periods of low electricity prices, power-to-heat technologies (P2H) are being integrated into the system.

P2H technology, which involves converting surplus electricity into thermal energy, has gained increasing attention as a viable solution for balancing energy supply and demand in renewable energy systems [6]. A promising application of this approach is the use of electric heaters to store excess electricity in high-temperature molten salts. The stored thermal energy can later be used for industrial heating, process steam generation, or electricity production in concentrated solar power (CSP) plants [8,9]. Therefore, the TES system not only plays a crucial role in bridging the gap between energy supply and demand but also increases the performance and reliability of energy systems [10]. In this context, analyzing thermal storage systems, including their thermal and structural compatibility, is becoming increasingly important due to the new operating conditions required by evolving energy challenges. The primary focus is on evaluating potential increases in operating temperatures to maximize system performance and productivity. To achieve this, it is essential to assess whether the storage medium and structural materials can withstand these enhanced conditions.

Molten salt is considered an excellent medium for heat transfer and energy storage in CSP plants due to its favourable thermophysical properties and lower cost compared to other media [11]. However, molten salt environments are highly aggressive and exhibit significant corrosive potential, which increases with temperature [12]. Traditionally, the most used molten salts have been nitrate-based [13], mainly due to their excellent thermophysical properties and thermal stability at operating temperatures of up to 400 °C. Nevertheless, in line with efforts to increase operating temperatures, it is necessary to explore other molten salts, such as those based on carbonate, fluoride, or chloride. Carbonate-based salts have higher melting points and viscosities than nitrate salts, but they offer thermal stability at much higher temperatures [14]. Meanwhile, chloride [15] and fluoride [16] salts are also under investigation; however, their primary disadvantage is their high corrosivity in the most widely studied materials.

Current configurations typically operate with molten nitrate salts, such as the binary mixture of NaNO_3_-NO_3_ (60-40 wt%), commonly known as solar salt [17], or ternary mixtures NaNO_3_-KNO_3_-NaNO_2_ (7-53-40 wt%), commercially known as Hitec [18]. However, the next generation of CSP plants is expected to operate at temperatures above 565 °C, close to the stability limit of nitrate salts [19]. Increasing the operating temperature in CSP plants enhances thermodynamic efficiency and lowers the levelized cost of energy (LCOE). It also allows integration with advanced cycles (e.g., supercritical CO_2_) and high-temperature thermal storage, while extending applicability to industrial process heat. Therefore, alternative molten salts need to be evaluated as potential storage media [20]. Although energy storage using molten salts has been widely studied, there are still several aspects of the materials that need to be investigated. These include the thermophysical properties of the salts, their thermal decomposition behavior under long-term thermal cycling, and the corrosion phenomenon.

On this matter, compatibility of the structural materials with heat transfer fluids (HTFs) and storage media is a crucial concern and a technical challenge. Corrosion of structural components significantly affects the lifespan and cost of CSP plants, making an accurate assessment of corrosion behavior essential. Among all components, storage tanks are the most critical elements, particularly the hot tank [21]. This equipment is especially vulnerable to corrosion due to prolonged exposure to high-temperature molten salts. To withstand such conditions, materials like SS304 and SS316, or those stabilized with titanium and niobium such as SS321 and SS347, are typically employed. However, as operating temperatures increase, corrosion rates rise significantly—even for these stainless steel alloys. Moreover, the electrification of heat with power-to-heat technologies introduces additional components, such as electric heaters. Understanding their behavior with respect to molten salt corrosion, particularly in potential hot spots, is essential. To this end, it is important to first review existing studies, where available, or identify the need for new research.

This review aims to examine the corrosion phenomena associated with the most commonly used molten salts, at both medium and high temperatures, when in contact with materials frequently employed in CSP installations. It also outlines strategies for mitigating corrosion, highlighting the importance of tailoring studies to address emerging challenges. The goal is to provide valuable insights for future research and development in the thermal storage sector, in line with current energy objectives.

## 2. Review of the Corrosion Process in CSP

For several decades, corrosion of molten salts in CSP has been widely studied, as evidenced by the large number of available publications. However, there is still much to know regarding the corrosion processes of metals with molten salts, especially those related to high temperatures and dynamic corrosion, and due to the lack of experimental data for many salt mixtures and materials studied.

Table 1 summarizes the most relevant articles included in this review. The review is structured by the type of corrosion test, static or dynamic, and the key factors analyzed.

### 2.1. Static Corrosion

Static corrosion refers to the processes that occur in environments where molten salt remains relatively still, such as within storage tanks in TES. Unlike dynamic corrosion, which involves mechanical effects caused by salt flow, static corrosion is primarily influenced by temperature, salt composition, and exposure time. This section explores the key mechanisms, factors, and findings associated with static corrosion in molten salts.

#### 2.1.1. Influence of Molten Salt Composition

At the microscopic level, corrosion in static molten salts often involves complex chemical reactions and interfacial interactions between the molten salts and metallic surfaces. Corrosion typically begins with oxidation processes, where the metal atoms at the surface lose electrons to form metal cations [22]. For example, in nitrate salts, the decomposition of nitrate ions (NO_3_) at high temperatures generates oxygen, which reacts with metallic components, forming oxides on the metal surface. However, many of these oxides, such as Cr_2_O_3_ in chromium-containing alloys, lack stability in molten salts and can dissolve back into the salt. This dissolution of protective oxide layers results in continuous corrosion, as the metal surface remains exposed. Carbonate salts also promote corrosion through oxidation but offer slightly better stability due to the formation of protective oxide layers in alloys with high chromium and nickel contents. In general, all studies conducted on carbonate molten salts show good performance at high temperatures [23]. In chloride-based molten salts, which are highly corrosive, corrosion involves both oxidation and chlorination [24]. Metal chlorides, such as FeCl_2_, form on the surface but are typically unstable and dissolve in the molten salt. This dissolution leads to further metal loss and significantly accelerates corrosion. Finally, fluoride-based salts exhibit high corrosion rates, which are significantly influenced by potential impurities within the components [25]. The validation conditions and specific molten salt compositions lead to a wide range of diverse and remarkable results.

Nitrate-based salts have been widely used in CSP plants, with solar salt being the most prominent example. Consequently, the number of studies related to the corrosion of this salt is extensive [17,26,27]. However, it is worthwhile to compare these findings with those of other ternary salts, such as Hitec, or other alternative mixtures. In this approach, Villada et al. [28] investigated the corrosion behavior of three nitrate salts with different compositions (solar salt, Hitec and a quaternary mixture with lithium, NaNO_3_-KNO_3_-NaNO_2_-LiNO_3_ (6-45-34-15 wt%)) in contact with the same material, SS304, at 550 °C and over two periods, 1000 and 2000 h. For solar salt, the oxide layer exhibited a thickness of 5.05 ± 0.56 µm at 1000 h and increased to 7.04 ± 0.97 µm at 2000 h, accompanied by a continuous mass gain. In contrast, for the Hitec mixture, corrosion products did not adhere to the surface, with a thickness of 13.42 ± 0.51 µm at 1000 h, decreasing to 4.84 ± 0.71 µm at the end of the test, likely due to detachment of the corrosion products. For the third salt, the oxide layer thickness was 4.60 ± 0.15 µm at 1000 h and grew to 10.96 ± 1.21 µm at 2000 h. This material demonstrated strong corrosion resistance when exposed to salts at 550 °C across all tested samples, suggesting its suitability for long-term use in a commercial thermal energy storage (TES) system.

Other authors have studied potential improvements, such as the incorporation of calcium nitrate (known as Hitec XL: NaNO_3_-KNO_3_-Ca(NO_3_)_2_ (7-45-48 wt%)). Fernandez et al. [29] compared the corrosion rates of solar salt and Hitec XL for low-Cr alloy steel (T22) and carbon steel (A1) at 390 °C. The rates decreased significantly from 0.11 µm/h for solar salt to 0.00075 µm/h for the Hitec XL mixture on carbon steel (A1). The analysis results confirm the better corrosion resistance of the tested steels, as evidenced by lower corrosion rates after exposure to HitecXL salt compared to those observed with solar salt. In general, it can be stated that, up to medium temperatures, the alloys commonly used in thermal plants exhibit good corrosion resistance when exposed to nitrate-based salts.

**Figure 1:**
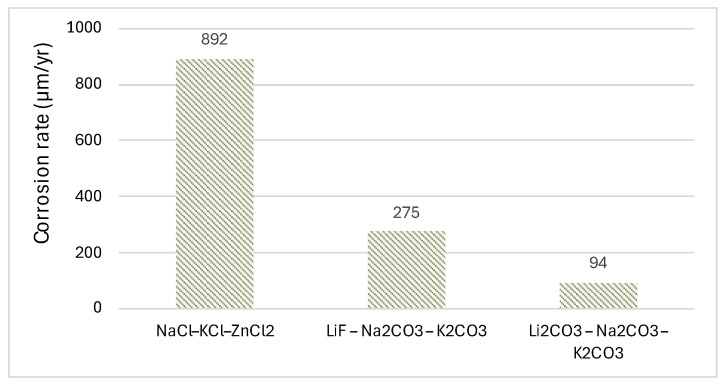
Influence of molten salt composition on SS316 alloy [30].

However, in line with current energy objectives, it is necessary to operate at higher temperatures, where nitrate salts approach their thermal instability limits. This necessitates the incorporation of alternative types of salts. In this context, Liu et al. [30] compared the eutectic mixtures NaCl-KCl-ZnCl_2_, LiF-Na_2_CO_3_-K_2_CO_3_, and Li_2_CO_3_-Na_2_CO_3_-K_2_CO_3_, as well as the alloys SS316, Hastelloy C276, Inconel-625 (In625), and Inconel-718 (In718) at 700 °C. Their results indicated that Zn salts are not viable due to their high corrosivity, while the superalloys C276 and In718 could form protective layers in Li salts. For example, in the specific case of SS316, the corrosion rate for the first salt (NaCl-KCl-ZnCl_2_) was nearly ten times higher than that of the carbon-based salt and three times higher than that of the remaining salt (second). Figure 1 illustrates the influence of the salt composition, as evaluated in the study by Liu et al. [30], for the SS316 alloy. Likewise, Na et al. [31] recently investigated the corrosion rate of a novel ternary salts mixture composed of NaNO_2-_KNO_3-_K_2_CO_3_ (44.55-54.45-1 wt%), which features a low melting point and a decomposition temperature above 660 °C. Researchers tested the salt by exposing stainless steel SS304 at 600 °C over various operating periods. The annual corrosion rates observed were 0.186 mm/yr, 0.095 mm/yr, 0.078 mm/yr, and 0.068 mm/yr after 250, 500, 750, and 1000 h of exposure, respectively. The corrosion products identified on the stainless steel surface included Fe_2_O_3_, Fe_3_O_4_, FeCr_2_O_4_, NaFeO_2_, and NiFe_2_O_4_. It would be necessary to extend the operating period beyond 1000 h to validate its potential use.

Regarding the use of fluoride-based salts, high corrosion rates limit their application to specific cases, such as molten salt reactors in the nuclear field. Hu et al. [32] analyzed the corrosion mechanism of a nickel-based alloy, GH3535, at 700 °C in molten LiF-NaF-KF salt. Currently, further validation is required to enable the use of these molten salts in thermal energy storage (TES) applications, which are the focus of this study.

Overall, nitrate-based salts demonstrate excellent corrosion resistance at medium temperatures when used with common alloys in CSP plants. Nevertheless, achieving higher operating temperatures requires consideration of alternative salt mixtures, such as those based on fluorides, chlorides, and carbonates. As current research shows, material compatibility at elevated temperatures remains a major challenge. Detailed analyses of salt formulations continue to highlight material compatibility issues at high operating temperatures. Moreover, for certain salts and high-temperature conditions, conclusive results are still lacking. As a result, the influence of temperature on corrosion behavior is currently being examined in greater depth to better understand and address these limitations.

#### 2.1.2. Influence of Temperature

**Figure 2:**
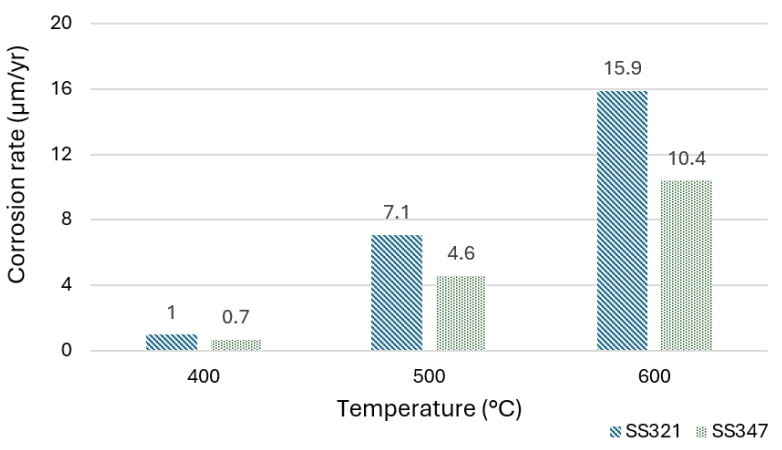
Influence of temperature on different alloys [35].

Temperature is a crucial factor influencing corrosion. The thermal stability of molten salts decreases with rising temperature, leading to accelerated corrosion rates. This trend has been confirmed by several experimental studies [33,34]. For example, Kruizenga and Gill [35] examined the corrosion of SS321 and SS347 at temperatures between 400 °C and 680 °C for up to 3000 h with solar salt. They found that corrosion rates varied significantly with temperature; at 400 °C and 500 °C, both alloys formed protective scales with parabolic kinetics, while at 600 °C, oxidation kinetics became linear with high rates of scale spalling. Corrosion products at 500 °C were mainly iron oxides with chromium depletion, while at 600 °C, sodium ferrite phases were found. SS347 performed better due to its higher Cr content. Corrosion rates remained below 16 µm/yr up to 600 °C but increased to 460 µm/yr at 680 °C. Figure 2 illustrates the results from the previous study, highlighting the effect of temperature on the behavior of the two investigated alloys (SS321 and SS347) in the 400–600 °C range. A similar study using the same molten salt, but different alloys, evaluated temperatures starting at 600 °C [36]. They evaluated high-temperature nickel-based alloys, In625 and Hastelloy 230, at 600 °C and 680 °C for 3000 h with solar salt, showing low corrosion rates at 600 °C and significant corrosion at 680 °C. The difference between the two temperatures is because the thermal stability limit of solar salt has been reached; the onset of salt decomposition can lead to the formation of more aggressive species, which accelerates the corrosion process. Concerning other nitrate-based salts, Zhu et al. [37] investigated the corrosion of SS316 in Hitec salt at 450 °C, 600 °C, and 680 °C. At 450 °C, the material demonstrated good corrosion resistance, forming a thin protective layer composed of Cr_2_O_3_ and iron-based oxides. However, a notable increase in corrosion rate was recorded at 600 °C and 680 °C. This increase is likely due to the dissolution of the protective Cr_2_O_3_ layer at these elevated temperatures.

Regarding the performance of other types of salts, Kondo et al. [38] performed static immersion tests on SS304 and SS316L in high-purity LiF–BeF_2_ at 500 °C and 600 °C for 1000 h. In both cases, the weight loss increased with temperature, resulting in corrosion rates of 3.26–5.42 µm/yr (SS316L) and 10.25–10.60 mm/yr (SS304), respectively. Although an increase in temperature is again associated with a higher corrosion rate, in these salts, due to their higher operating temperatures, no significant change was observed, unlike in nitrate-based salts.

In conclusion, temperature is a key parameter influencing corrosion in all molten salt systems.

#### 2.1.3. Influence of Alloy Composition

Historically, the influence of alloy composition has been a widely studied factor. For this reason, certain alloys are more commonly used in tanks, pipes, and other equipment in CSP plants, as they aim to strike a balance between mechanical–structural stability and economic considerations. Among the primary structural alloys used in the industrial field, Fe-based and Ni-based alloys are the most common. As such, these alloys are examined as examples to demonstrate how alloying elements influence the corrosion resistance of these materials.

**Figure 3:**
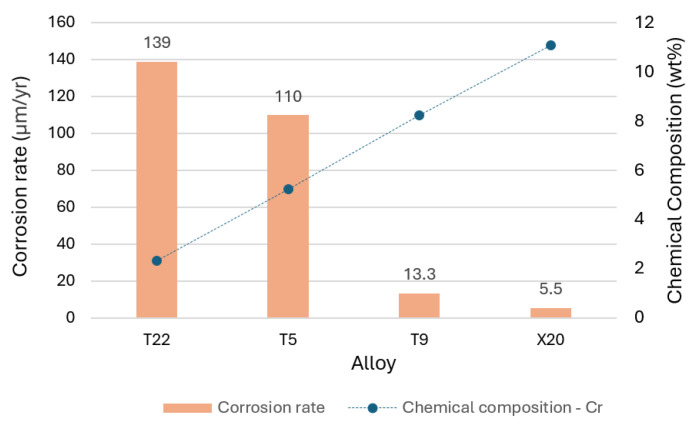
Influence of alloy composition, specifically chromium content [40].

Initial studies on the corrosion behavior of alloys in molten salts were conducted by Slusser et al. [39]. They examined high Ni Fe-based alloys (Nicrofer 3718, 253 MA, SS330, and SS310) and Ni-based alloys (In600, In601, Hastelloy N, In800, and pure Ni 200) in molten salts at temperatures between 510 °C and 705 °C. The study found that all alloys suffered corrosion, with Ni-based alloys containing 15–20% Cr performing better, while iron-based alloys without Ni showed little resistance at high temperatures. Cheng et al. [40] reported similar findings. Corrosion testing was conducted by immersing Cr–Mo steels with varying chromium contents 0 wt% (SB450), 2.34 wt% (T22), 5.25 wt% (T5), 8.25 wt% (T9), and 11.08 wt% (X20), in molten eutectic salt LiNO_3_-NaNO_3_-KNO_3_ at 550 °C for durations of 250, 500, and 1000 h under a nitrogen cover gas. A strong dependence of the corrosion rate on chromium content was observed after 1000 h of operation. The corrosion rates ranged from 153 μm/yr for 0 wt% Cr, 139 μm/yr for 2.34 wt%, 110 μm/yr for 5.25 wt%, 13.3 μm/yr for 8.25 wt%, and 5.5 μm/yr for 11.08 wt%. These results showed that the corrosion resistance of the alloy increases with increasing Cr content in the alloy (Figure 3). The corrosion layer’s thickness decreased with increasing Cr content.

To evaluate the effect of nickel content in alloys, Sun et al. [41] studied a molten ternary eutectic NaCl-KCl-MgCl_2_ (33-21.6-45.4 mol%) in contact with SS316 and seven Ni-based alloys (In617, Haynes242, Hastelloy C276, Hastelloy C22, In600, In625, Haynes 230), finding that SS316 exhibited lower corrosion resistance than Ni-based alloys. Among the nickel-based alloys, corrosion resistance improved in the following order: Ni-Fe-Cr < Ni-W-Cr < Ni-Mo-Cr. These results demonstrate the influence of alloy composition, with an improvement in the behavior of alloys with a higher percentage of nickel in their composition. Similar results were recently obtained with a chlorine-based salt, NaCl-KCl-CaCl_2_ (24.5-8.2-67.3 wt%) [42]. The corrosion mechanism was analyzed at 600 °C in contact with three different materials: TP347H stainless steel, Haynes230 (HA230), and In625 alloys. The average corrosion rates were 2383.6 μm/yr for TP347H, 487.6 μm/yr for HA230, and 5437.5 μm/yr for In625. The superior corrosion resistance of the HA230 alloy is attributed to its higher nickel (Ni) and tungsten (W) content.

Once the influence of alloy composition on the corrosion phenomenon has been established, this final section will focus on the most used alloys in CSP installations, as SS304, SS316L, and SS347H. Goods and Bradshaw [43] investigated isothermal corrosion in solar salt at 570 °C for 7000 h for austenitic stainless steels 304 and 316. The SS316 and SS304 showed metal losses between 6 and 15 µm/yr, respectively. For the binary nitrate-based salt, Gomes et al. [34] also evaluated the corrosion behavior after exposure for 3000 h at 550 °C to SS321H and SS316L. The measured corrosion rates were 9 µm/yr for SS321H and 8.6 µm/yr for SS316L, indicating relatively low material degradation under these conditions.

The effect of other salt mixtures on the most used alloys has also been evaluated, although to a lesser extent than solar salt, as evidenced by the smaller number of studies. Sah et al. [44] studied the corrosion behaviors of SS310S, SS316L, and SS304 in carbonate molten salt (Li_2_CO_3_-Na_2_CO_3_-K_2_CO_3_) at 650 °C under the atmosphere of CO_2_–2%O_2_ and found that the corrosion-resistance of the stainless steels increased with the increase of Cr and Ni content in the matrix. The corrosion resistance of SS310S was better than those of SS316L and SS304. The previously analyzed mixture was also studied by Grosu et al. [45]. They investigated the corrosion resistance of austenitic stainless steels SS310 and SS347 at 600 °C. After exposure to the molten salt, the SS347 specimen was covered by Cr_2_NiO_4_, CrNiO_4_, and Fe_2_O_3_, and the scale was found to have a highly non-homogeneous thickness with an average value of (8.2 ± 4.6) μm. For SS310, the scale thickness was also found to be very non-homogeneous, and the average thickness was found to be (8.1 ± 5.8) μm. Finally, Wang et al. [46] performed static tests at 500 °C up to 1440 h in a ternary salt, KNO_3_-LiNO_3_-Ca(NO_3_)_2_ (67.2-13.5-19.3 wt%) for SS304 and SS316L, finding corrosion rates of 0.031 mm/yr and 0.014 mm/yr, respectively.

Therefore, the influence of alloy composition on the corrosion mechanism has been demonstrated. Key factors such as the nickel and chromium content have proven to play a critical role in determining corrosion resistance. Tungsten, on the other hand, has shown some influence, albeit to a lesser extent, mainly contributing to high-temperature strength and structural performance. Additionally, the performance of the most used alloys in CSP facilities has been thoroughly evaluated, highlighting their strengths and limitations under high-temperature salt exposure. Possible improvement measures, including alloying modifications and protective oxide layer formation, have also been explored to enhance long-term material stability in these demanding environments.

#### 2.1.4. Influence of Impurities

**Figure 4:**
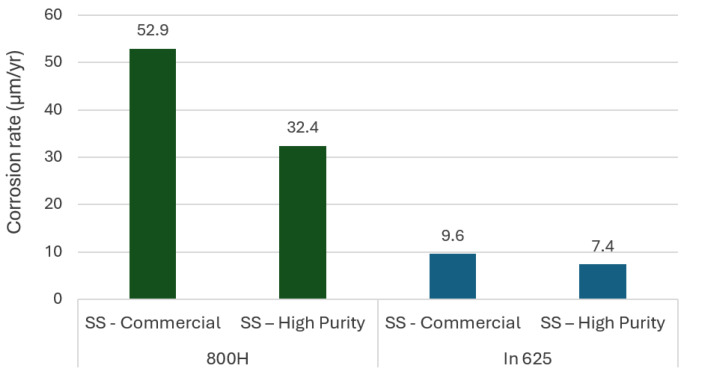
Influence of impurities in solar salt (commercial, high-purity) on corrosion resistance [50].

The presence of impurities, such as Mg, Ca, and chloride ions, within nitrate salts is a major contributor to increased corrosivity. Cordero et al. [47] analyzed molten nitrate salts and noted that equilibration between NO_2_ and NO_3_ occurs at 220 °C, with NO_3_ decreasing as temperature rises to 550 °C. This shift leads to increased NO_2_ formation and a corresponding reduction in the thermal stability of the molten salt. At high temperatures, molten nitrates react with CO_2_ to form carbonates, and CO_2_ interacts with impurities (Ca, Mg) to form insoluble carbonate compounds, further limiting the thermal stability and corrosion resistance of the salts. Similar results were obtained by Prieto et al. [48]. They found that the impurity Mg(NO_3_)_2_ was the main source of NOx emissions in solar salts during plant start-up, due to its thermal decomposition during the melting process. This compound acts as a strong oxidizing agent and may significantly accelerate corrosion. In another study, Prieto et al. [49] evaluated the corrosion of A516Gr70 carbon steel samples exposed to solar salts containing various chloride concentrations, observing the direct effect of this element on corrosion rates. They observed corrosion rates ranging from 83.2 µm/yr for salt without chlorine content to 987.3 µm/yr for mixtures with a chlorine content of 1.8%. The effect of impurities was also studied by the same authors using other alloys [50]. They studied the alloys 800H and In625 in high-purity and commercial solar salt (SS) at 565 °C for up to 1470 h. As shown in Figure 4, the corrosion rate was higher in commercial salt, and In625 consistently exhibited better corrosion resistance than 800H under all conditions tested.

Similarly, Goods and Bradshaw [43] investigated isothermal corrosion in seven mixtures of NaNO_3_ and KNO_3_ with varying impurity concentrations. Tests were conducted at 570 °C for 7000 h for SS304 and SS316, and at 316 °C for A36 steel. The corrosion rates for SS316 and SS304 corresponded to metal losses of 6–15 µm/yr. For SS304, the effect of increasing chloride concentration was evident, as the weight loss rate increased with higher chloride levels. In contrast, SS316 showed corrosion rates largely unaffected by impurity levels in the molten salt. A36 steel showed relatively high tolerance to the impurities commonly found in commercial-grade alkali nitrates. The influence of deliberate additions of chloride to a high-purity nitrate mixture was not observed. In more recent studies, Oskay et al. [51] replicated investigations on the influence of impurities in solar salt using different alloys (X20, Alloy 800H, and HA230) at 600 °C. The original salt mixture contained 131 ppm of Cl^−^ and 59 ppm of SO_4_^2−^ impurities. To study the effects of higher impurity levels, 500 and 1000 ppm of each ion were introduced by adding the appropriate amounts of NaCl and Na_2_SO_4_ to the mixture. Given the relatively low corrosion resistance of X20 compared to Alloy 800H and HA230, the influence of impurities on corrosion kinetics was observed in all cases, with different trends depending on the alloy.

In conclusion, the presence and concentration of impurities, such as chlorides and sulphates, play a significant role in influencing corrosion kinetics in molten salt environments. The impact of these impurities can vary not only in magnitude but also in trend depending on the alloy’s composition and microstructure. Therefore, understanding the specific interactions between impurities and material surfaces is essential for selecting and designing corrosion-resistant materials for high-temperature molten salt applications.

#### 2.1.5. Influence of Atmosphere

The composition of the surrounding atmosphere is a key factor in many processes, as it can significantly influence the outcome. In particular, the presence or absence of an inert atmosphere plays a crucial role.

In this context, Xu et al. [52] analyzed the behavior of In625 alloy in contact with a ternary chloride-based salt mixture (NaCl-CaCl_2-_MgCl_2_) at 600 °C for 500 h, using air and nitrogen as cover gases. They also varied the MgCl_2_ content in the salt mixture. The study found that the presence of O_2_, H_2_O, and MgOH^+^ significantly influenced the alloy’s performance, depending on the atmosphere. In air, increasing the MgCl_2_ content accelerated alloy dissolution, as reactions between H_2_O and O_2_ generated MgOH^+^ impurities that promoted corrosion. However, the formation of corrosion products, such as MgO and MgCr_2_O_4_, provided a protective barrier against further degradation. In contrast, when the salt lacked Mg^2+^, no protective corrosion products formed due to the absence of MgOH^+^, resulting in low corrosion resistance. In a nitrogen atmosphere, thermally induced stresses caused cracking of the corrosion layer on the alloy surface. The MgO and MgCr_2_O_4_ scales failed to offer sufficient protection, allowing corrosive species to penetrate the metal matrix and propagate along grain boundaries, ultimately resulting in grain detachment.

Other studies have reported similar findings. Bell et al. [53] investigated the corrosion behavior of SS316L in molten NaCl-Na_2_CO_3_ under different atmospheres. They exposed the samples to molten salts at 650 °C in both argon and air for up to 900 h. In air, the oxide scale thickness increased significantly from 30 µm at 120 h to 280 µm at 900 h, while in argon, it grew more slowly, from 15 µm to 45 µm over the same period. Analysis revealed that the scale contained Fe^2+^ and Fe^3+^ species, whereas only Cr^2+^ was detected, suggesting that Cr^3+^ ions had dissolved into the molten salt. Corrosion rates were notably higher in air than in argon. Additionally, the microstructure of the corrosion layer varied with atmospheric conditions, indicating distinct corrosion mechanisms under oxidizing and inert conditions.

In summary, atmospheric conditions play a crucial role in the corrosion behavior of metal alloys exposed to molten salts. Oxidizing environments, such as air, significantly accelerate corrosion due to the presence of reactive species like oxygen, water vapor, and hydroxide impurities, which promote the formation of corrosive compounds and lead to thicker oxide scales. Inert atmospheres, such as nitrogen or argon, generally reduce corrosion rates but may still result in cracking issues due to thermal stress. These observations underscore the importance of atmosphere control in high-temperature molten salt environments to enhance alloy durability.

#### 2.1.6. Additional Key Factors

Finally, the effects of several other important parameters are analyzed, including exposure time, exposure type (continuous or intermittent), and the impact of humidity.

Regarding the exposure time, Brashaw et al. [54] analyzed the corrosion behavior of SS316L, SS316, and SS304 at 565 °C using solar salts with different impurities. They increased the exposure duration and applied two test types: thermal cycling (7.5 h at 565 °C followed by cooling to 95 °C for 15 min, then repeating the cycle) and isothermal exposure. Thermal cycling led to moderately higher corrosion rates compared to isothermal immersion. SS316 showed a 25% to 50% increase in corrosion under thermal cycling, depending on the chloride content of the salt. SS316L was the least affected, with no more than a 25% increase over 4000 h. SS304 showed up to 50% higher corrosion rates when thermally cycled, again influenced by the chloride content and impurities previously analyzed.

Prieto et al. [17] also investigated the influence of exposure mode, testing carbon and low-alloy steels, such as A516Gr70, A387Gr11, and A387Gr5, Cr–Mo steel A387Gr9, and austenitic stainless steels including SS304L, SS316L, and SS347. Their study evaluated the effects of both temperature and exposure duration, as well as the differences between continuous and intermittent exposure. They found that intermittent exposure led to increased corrosion rates in almost all cases (as detailed in Table 1). The alloys were ranked in terms of increasing corrosion resistance as follows: A387Gr5~A387Gr11 < A516Gr70 < A387Gr9 < SS304L~SS316L~SS347.

Regarding the influence of humidity, Ouyang et al. [55] investigated the corrosion characteristics of several selected alloys (Hastelloy-N, Hastelloy-B3, HA242, HA263) at 600 °C and 700 °C in LiF-NaF-KF (46.5-11.5-42 mol%) molten salts with moisture content ranging from 3.19 wt% to 1.91 wt%. In this work, they discovered that the water content in this molten salt had a significant effect on the corrosion of Ni-based alloys at high temperatures. Increasing water content accelerated the reaction between H_2_O and F^−^, which increased the activity of O^2−^ and HF in molten salt, leading to increased corrosion.

Similar results were obtained by Grosu et al. [56], who evaluated the effect of humidity on the corrosion behavior of different alloys, A516 Gr70, SS304, and SS316, when exposed to Hitec XL molten salt. For the carbon steel A516 Gr70, the influence of humidity became more significant as the exposure time increased, indicating a progressive deterioration. In contrast, although humidity also affected the stainless steels SS304 and SS316, its impact was less pronounced and remained relatively stable over time. These findings highlight the varying sensitivity of different alloys to moisture in high-temperature salt environments.

This review underscores the importance of operating conditions, salt composition, and container materials in determining corrosion behavior. Although static immersion tests have provided valuable insights, there is a growing need for studies under actual operating conditions to better understand and mitigate corrosion in CSP systems.

### 2.2. Dynamic Corrosion

Dynamic corrosion refers to the corrosion processes occurring under flowing molten salt conditions, as found in pipelines, pumps, and heat exchangers within thermal energy storage (TES) systems. These conditions introduce additional factors, such as flow velocity and shear stress, which can accelerate corrosion by enhancing the mechanical wear on oxide layers and facilitating molten salt access to metal surfaces. Experience in plant operations has shown that corrosion behavior is more severe under dynamic conditions than in static experiments [57]. However, as this review will demonstrate, the number of dynamic experiments and the diversity of salts and alloys investigated is considerably lower than in static testing.

The most relevant studies are summarized in Table 1. They are listed as a type of dynamic test, with flow rate identified as one of the most influential factors. Several studies have compared results obtained under static and dynamic operating conditions. García-Martín et al. [58] studied A516 carbon steel under dynamic conditions at a temperature of 500 °C for 100 h. Their system included a molten salt tank with circulation to a testing chamber at a flow rate of 0.2 m/s. Corrosion rates were up to 55% higher in dynamic conditions compared to static conditions. Cross-sectional micrographs also showed more severe corrosion under dynamic testing, with an oxide layer thickness of 31.0 µm compared to 23.3 µm in static tests. Additionally, the oxide layer formed during dynamic testing was more compact, likely due to the tangential force of the flowing salt removing loosely adherent oxide layers.

Fernández et al. [59] conducted a comparative study on the corrosion behavior of stainless steel 316 (SS316) under static and dynamic conditions (flow rate molten salt 0.3 m/s) using a lithium-containing molten salt mixture, LiNO_3_-KNO_3_-NaNO_3_ (30-57-13 wt%), at 390 °C for 1000 h. Their results showed that corrosion rates in dynamic conditions were more than twice those observed in static tests. The same authors also employed the pilot plant with the same molten salt composition, substituting A516 for SS316. Under the same dynamic conditions (0.3 m/s, 390 °C), a corrosion rate of 15 µm/yr was obtained [59]. Mallco et al. [60] also investigated T91 steel in the same ternary salt mixture at 550 °C for up to 650 h. They reported a marked increase in corrosion rates after 400 h under dynamic conditions, indicating that erosion, alongside corrosion, contributed to material degradation.

Recently, Wang et al. [61] highlighted the importance of conducting dynamic corrosion tests. They studied the corrosion mechanisms of the NaNO_3_-Na_2_CO_3_ salt mixture at 600 °C using SS304, SS316L, and SS347H alloys over a duration of 1000 h. The results indicated that, at a flow rate of 2 m/s, the corrosion rates for SS304, SS316L, and SS347H were 0.0217 mm/yr, 0.0122 mm/yr, and 0.0076 mm/yr, respectively. These values were 3.8, 3.4, and 2.2 times higher than those observed under static conditions, emphasizing the significant impact of flow-induced effects on corrosion behavior.

#### Influence of Velocity

Flow velocity is a critical factor in dynamic corrosion, as increased velocity enhances the erosive aspect of the process. Studies demonstrate that corrosion rates rise with increasing flow velocities, likely due to the mechanical removal of protective oxide layers and enhanced transport of corrosive species to the metal surface.

In this context, several studies have aimed to highlight the influence of flow rate on corrosion behavior. Zhang et al. [62] analyzed the corrosion of SS316 and SS321 in solar salt at 565 °C under flow rates of 1 m/s, 2 m/s, and 3 m/s. Their findings showed that the velocity of the molten salt had a significant impact on the corrosion processes of both SS316 and SS321. Recent studies, such as those by Yang et al. [63], investigated the corrosion of SS304 (with and without welding) and Q275 alloy at 500 °C in solar salt. The tests involved immersing samples in molten salts stirred by paddles, with flow rates ranging from 1 m/s to 2.5 m/s, for up to 500 h. The welded zones showed increased susceptibility to intergranular corrosion. The corrosion rates were ranked as follows: SS304 (without welds) < SS304 (with welds) < Q275, with higher flow velocities correlating with increased corrosion rates. In quaternary nitrate–nitrite mixtures, Ma et al. [64] investigated the dynamic corrosion of SS316L stainless steel at 565 °C with varying flow rates (0.6 m/s, 1.3 m/s, and 2 m/s) for 1000 h. The corrosion rate increased with higher flow rates, likely due to the erosive effect on the oxide layer.

These findings underscore the urgent need for further research focused on dynamic corrosion testing under realistic operating conditions. Compared to static tests, data on dynamic corrosion are still limited, particularly for high-temperature applications and across a range of flow velocities. Future studies should address non-isothermal conditions, paying special attention to hot spots that commonly develop in electric heaters (P2H technologies), where thermal gradients may intensify erosion–corrosion effects. Despite existing studies and recent technological advances, there is still a lack of comprehensive data for various alloys and for temperatures exceeding standard operating conditions. Understanding how these factors interact is critical to ensuring the durability and efficiency of thermal energy storage (TES) systems. As a result, there is growing interest in evaluating and developing effective corrosion mitigation strategies.

**Table 1:** Review of studies on the corrosion phenomenon of molten salts and materials.

Material	Molten Salt	Type	Working Temperature (°C)	Exposition Time (h)	Flow Rate (m/s)	Corrosion Rate (µm/yr)	Conclusion	Ref.
SS304	Solar Salt	St *	550	1000	-	44.24	Influence of molten salt type: Hitec salt generally exhibits a higher corrosion rate compared to binary salt. However, under prolonged exposure, Hitec ternary salt may degrade over time, gradually transitioning toward the composition of a binary salt.	[28]
SS304	Solar Salt	St *	550	2000		30.84		
SS304	Hitec	St *	550	1000		117.56		
SS304	Hitec	St *	550	2000		21.20		
SS304	KNO_3_ + NaNO_2_ + LiNO_3_ + NaNO_3_	St *	550	1000		40.30		
SS304	KNO_3_ + NaNO_2_ + LiNO_3_ + NaNO_3_	St *	550	2000		48.00		
SS316L	Solar Salt	St *	565	1187	-	21.00		[65]
A1	Hitec XL	St *	390	2000	-	6.57	Influence of molten salt composition and alloy.	[29]
A1	Solar Salt	St *	390	2000		970.61		
T22	Hitec XL	St *	390	2000		3.85		
T22	Solar Salt	St *	390	2000		70.96		
SS316	NaCl-KCl-ZnCl_2_	St *	700	504	-	1700	Influence of molten salt composition and alloy.	[30]
C276	NaCl-KCl-ZnCl_2_	St *	700	504		668		
In625	NaCl-KCl-ZnCl_2_	St *	700	504		447		
In718	NaCl-KCl-ZnCl_2_	St *	700	504		962		
C276	Li_2_CO_3_-Na_2_CO_3_-K_2_CO_3_	St *	700	504		100		
In625	Li_2_CO_3_-Na_2_CO_3_-K_2_CO_3_	St *	700	504		936		
In718	Li_2_CO_3_-Na_2_CO_3_-K_2_CO_3_	St *	700	504		146		
SS316	LiF-Na_2_CO_3_-K_2_CO_3_	St *	700	336		796		
C276	LiF-Na_2_CO_3_-K_2_CO_3_	St *	700	504		1304		
In625	LiF-Na_2_CO_3_-K_2_CO_3_	St *	700	504		2097		
SS304	NaNO_2_-KNO_3_-K_2_CO_3_	St *	600	250	-	186	Influence of exposure time.	[31]
SS304	NaNO_2_-KNO_3_-K_2_CO_3_	St *	600	500		95		
SS304	NaNO_2_-KNO_3_-K_2_CO_3_	St *	600	750		78		
SS304	NaNO_2_-KNO_3_-K_2_CO_3_	St *	600	1000		68		
SS321	Solar Salt	St *	400	3000	-	1	Influence of temperature and alloy.	[35]
SS321	Solar Salt	St *	500	3000		7.1		
SS321	Solar Salt	St *	600	3000		15.9		
SS321	Solar Salt	St *	680	1000		460		
SS347	Solar Salt	St *	400	3000		0.7		
SS347	Solar Salt	St *	500	3000		4.6		
SS347	Solar Salt	St *	600	3000		10.4		
SS347	Solar Salt	St *	680	1000		447		
HA230	Solar Salt	St *	600	3000	-	23.6	Influence of temperature and alloy.	[36]
In625	Solar Salt	St *	600	3000		16.8		
HA230	Solar Salt	St *	680	1000		688		
In625	Solar Salt	St *	680	1000		694		
SS304	LiF-BeF_2_	St *	500	1000	-	10.25	Influence of temperature and alloy.	[38]
SS304	LiF-BeF_2_	St *	600	1000		10.6		
SS316L	LiF-BeF_2_	St *	500	1000		3.26		
SS316L	LiF-BeF_2_	St *	600	1000		5.42		
Nicrofer 3718, 253 MA, SS330, SS310, IN600, IN601, Hastelloy N, In800 and Ni200	Solar Salt	St *	510–705	-	-	-	Alloys with higher Cr content have greater resistance to corrosion. Demonstrated influence of temperature, very severe corrosion effect with temperatures above 650 °C.	[39]
SB450	LiNO_3_-NaNO_3_-KNO_3_	St *	550	1000	-	153	Influence of alloy composition.	[40]
T22	LiNO_3_-NaNO_3_-KNO_3_	St *	550	1000		139		
T5	LiNO_3_-NaNO_3_-KNO_3_	St *	550	1000		110		
T9	LiNO_3_-NaNO_3_-KNO_3_	St *	550	1000		13.3		
X20	LiNO_3_-NaNO_3_-KNO_3_	St *	550	1000		5.5		
TP347H	NaCl-KCl-CaCl_2_	St *	600	400	-	2383.6	Influence of alloy composition.	[42]
HA230	NaCl-KCl-CaCl_2_	St *	600	400		487.6		
In625	NaCl-KCl-CaCl_2_	St *	600	400		5437.5		
SS304	Solar Salt	St *	570	7000	-	6	Influence of alloy composition.	[43]
SS316	Solar Salt	St *	570	7000		15		
SS321H	Solar Salt	St *	550	3000	-	9	Influence of alloy composition.	[34]
SS316L	Solar Salt	St *	550	3000		8.6		
SS310S	Li_2_CO_3_-Na_2_CO_3_-K_2_CO_3_	St *	650	24	-	3500	Influence of alloy composition.	[44]
SS316L	Li_2_CO_3_-Na_2_CO_3_-K_2_CO_3_	St *	650	24		2900		
SS304	Li_2_CO_3_-Na_2_CO_3_-K_2_CO_3_	St *	650	24		500		
SS310	Li_2_CO_3_-Na_2_CO_3_-K_2_CO_3_	St *	600	600	-	118.26	Influence of alloy composition.	[45]
SS347	Li_2_CO_3_-Na_2_CO_3_-K_2_CO_3_	St *	600	600		119.72		
A516Gr70	Solar Salt	St *	400	1504	-	83.2	Influence of chloride content impurities.	[49]
A516Gr70	Solar Salt + 0.7% Cl-	St *	400	1504		587.8		
A516Gr70	Solar Salt + 1.8% Cl-	St *	400	1504		987.3		
800H	Solar Salt-Commercial	St *	565	1470	-	52.9	Influence of impurities.	[50]
800H	Solar Salt-High-Purity	St *	565	1470		32.4		
In625	Solar Salt-Commercial	St *	565	1470		9.6		
In625	Solar Salt-High-Purity	St *	565	1470		7.4		
X20	Solar Salt	St *	600	1000	-	113.88	Influence of impurities.	[51]
X20	Solar Salt + (500 ppm Cl + 500 ppm S)	St *	600	1000		148.92		
X20	Solar Salt + (1000 ppm Cl + 1000 ppm S)	St *	600	1000		613.2		
800H	Solar Salt	St *	600	1000		105.12		
800H	Solar Salt + (1000 ppm Cl + 1000 ppm S)	St *	600	1000		70.08		
HA230	Solar Salt	St *	600	1000		65		
HA230	Solar Salt + (500 ppm Cl + 500 ppm S)	St *	600	1000		43.8		
HA230	Solar Salt + (1000 ppm Cl + 1000 ppm S)	St *	600	1000		65		
A516Gr70	Solar Salt	St *	400	1632	-	27.6 (C)	Influence of exposure type, continuous (C) versus intermittent (I), as well as operating temperature.	[17]
A516Gr70	Solar Salt	St *	400	1632		238 (I)		
A516Gr70	Solar Salt	St *	280–325	1680		8 (C)		
A516Gr70	Solar Salt	St *	280–325	1680		18 (I)		
A387Gr11	Solar Salt	St *	400	1632		19 (C)		
A387Gr11	Solar Salt	St *	400	1632		420 (I)		
A387Gr11	Solar Salt	St *	280–325	1680		8.02 (C)		
A387Gr11	Solar Salt	St *	280–325	1680		14.8 (I)		
A387Gr5	Solar Salt	St *	400	1632		1.63 (C)		
A387Gr5	Solar Salt	St *	400	1632		464 (I)		
A387Gr5	Solar Salt	St *	280–325	1680		2.25 (C)		
A387Gr5	Solar Salt	St *	280–325	1680		1.98 (I)		
A387Gr9	Solar Salt	St *	400	1632		0.45 (C)		
A387Gr9	Solar Salt	St *	400	1632		1.47 (I)		
A387Gr9	Solar Salt	St *	280–325	1680		0.72 (C)		
A387Gr9	Solar Salt	St *	280–325	1680		0.55 (I)		
A304L	Solar Salt	St *	400	1632		0.41 (C)		
A304L	Solar Salt	St *	400	1632		0.61(I)		
A304L	Solar Salt	St *	280–325	1680		0.11 (C)		
A304L	Solar Salt	St *	280–325	1680		0.25 (I)		
A316L	Solar Salt	St *	400	1632		0.14 (C)		
A316L	Solar Salt	St *	400	1632		0.44 (I)		
A316L	Solar Salt	St *	280–325	1680		0.01 (C)		
A316L	Solar Salt	St *	280–325	1680		0.11 (I)		
A347	Solar Salt	St *	400	1632		0.77 (C)		
A347	Solar Salt	St *	400	1632		0.36 (I)		
A347	Solar Salt	St *	280–325	1680		0.32 (C)		
A347	Solar Salt	St *	280–325	1680		0.48 (I)		
A516	LiNO_3_-KNO_3_-NaNO_3_	Dy *	390	1000	0.3	15		[66]
SS304	NaNO_3_-Na_2_CO_3_	Dy *	600	1000	2	21.7	Influence of alloy composition.	[61]
SS316L	NaNO_3_-Na_2_CO_3_	Dy *	600	1000	2	12.2		
SS347H	NaNO_3_-Na_2_CO_3_	Dy *	600	1000	2	7.6		
SS316	Solar Salt	Dy *	565	1000	1	4.5	Influence of flow rate and alloy composition.	[62]
SS321	Solar Salt	Dy *	565	1000	1	5.1		
SS316	Solar Salt	Dy *	565	1000	2	4.6		
SS321	Solar Salt	Dy *	565	1000	2	5.4		
SS316	Solar Salt	Dy *	565	1000	3	5.1		
SS321	Solar Salt	Dy *	565	1000	3	5.7		
304SS	Solar Salt	Dy *	500	500	2.5	59	Influence of alloy composition and welding effects	[63]
304SS (With welds)	Solar Salt	Dy *	500	500	2.5	66		
Q275	Solar Salt	Dy *	500	500	2.5	93		

* St: static; * Dy: dynamic.

## 3. Corrosion Mitigation Strategies

Mitigating corrosion in thermal energy storage (TES) systems is essential to improve the durability of materials exposed to molten salts, particularly under the harsh conditions of concentrated solar power (CSP) plants. This section examines several mitigation strategies aimed at specific corrosion mechanisms, such as the destabilization of oxide layers by impurities, high-temperature erosion–corrosion, and chemical degradation in chloride environments. The strategies include molten salt purification, corrosion inhibitors, the use of nanoparticles, alumina-forming alloys, and protective coatings. Table 2 summarizes all relevant studies that discuss the application of the reviewed strategies.

### 3.1. Molten Salt Purification

Purification of molten salts effectively mitigates corrosion by reducing impurities, which are among the main contributors to corrosion in TES systems. Common impurities, such as magnesium, calcium, and chloride ions, compromise the stability of oxide layers on metals, accelerating degradation by promoting the formation of soluble compounds. Through purification, these reactive ions are minimized, enhancing the corrosion resistance of both the salt and the metal.

Prieto et al. [49] analyzed the effect of impurities on the corrosion behavior of solar salts in various alloy types. Their findings indicated that a significant reduction in chloride levels resulted in a marked decrease, by more than half, in the corrosion rate of carbon steel. With regard to other types of salts, Gong et al. [67] also studied the purification of molten salts, specifically a chloride-based mixture (MgCl_2_-KCl-NaCl) using an optimized Mg-additive method. This study involved two iron-based alloys, SS310 and In800H, which were exposed to salt for 2000 h, providing valuable insights into long-term corrosion performance under high-temperature conditions. In purified molten salt at 700 °C under an argon atmosphere, the corrosion rates of In800H and SS310 were measured at 7.6 ± 1.6 µm/yr and 4.9 ± 2.2 µm/yr, respectively.

Therefore, salt purification decreases corrosion by minimizing the reactive impurities that destabilize oxide layers on metal surfaces. Despite their effectiveness, purification methods face challenges in achieving and sustaining high-purity salts in large-scale TES systems, necessitating continuous monitoring and control.

### 3.2. Addition of Nanoparticles

Nanoparticle additives, particularly alumina (Al_2_O_3_) and silica (SiO_2_), present a promising strategy for reducing corrosion by promoting stable protective layers on metal surfaces. Upon addition to molten salts, nanoparticles diffuse onto metal surfaces, forming oxide layers that serve as protective barriers against corrosive agents.

**Figure 5:**
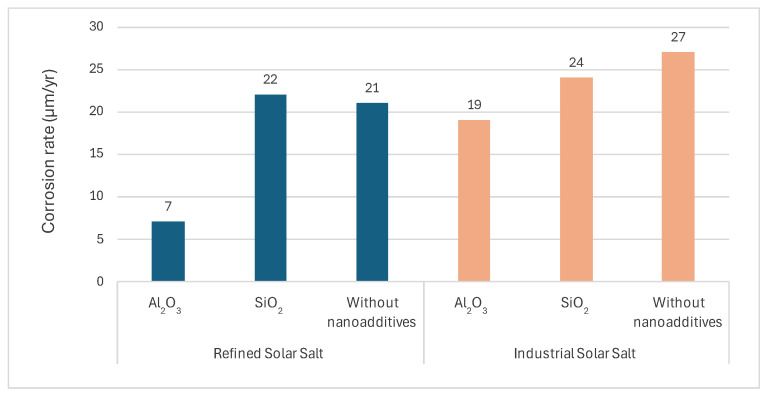
Effect of SiO_2_ and Al_2_O_3_ additions on the corrosion behavior of refined/industrial solar salt [69].

Numerous studies have reported noteworthy findings regarding the corrosion-reducing effects of nanoparticle additives. Camacho et al. [68] analyzed a model to understand the physical and chemical interactions between nanoparticles and structural materials, specifically carbon steel at 390 °C. The results indicate that the nanoadditive diffusion plays a key role in the formation of a protective layer, even without the presence of molten salt. This implies that a high-temperature pre-treatment of construction materials with nanoparticles may provide an effective alternative method of corrosion protection. Similarly, Fernández et al. [69] conducted an extensive corrosion characterization at 565 °C on two grades of solar salt (industrial and refined purity) with 1 wt% Al_2_O_3_ and SiO_2_ nanoparticles. The lowest corrosion rate (7 µm/yr) was observed in refined solar salt with 1 wt% Al_2_O_3_ nanoparticle, attributed to the formation of a protective Al_2_O_3_ layer at the steel–salt interface. The addition of SiO_2_ to refined salt did not show a significant effect compared to the case without additives. However, in the case of industrial solar salt, the effect of adding both alumina and silica was clearly observed. The results are presented in Figure 5.

Nithiyanantham et al. [12] also found that adding Al_2_O_3_ and SiO_2_ to binary nitrate salt significantly reduced the corrosion rates of carbon steel at 390 °C. After 1500 h of immersion, the corrosion layer was approximately three times thinner compared to that formed in the fluid without nanoparticles.

In other studies, Han et al. [70] have used Al_2_O_3_ nanoparticles in chloride mixtures (NaCl-KCl-MgCl_2_) to enhance the corrosion resistance of In625 at temperatures between 500 and 700 °C for up to 100 h in static immersion. These results indicate that nanoparticles can act as corrosion inhibitors by forming protective layers such as MgCr_2_O_4_ and other metallic oxides. However, Grosu et al. [71] observed that adding alumina and silica nanoparticles at 1 wt% to NaNO_3_-KNO_3_-Ca(NO_3_)_2_ (15-43-42 wt%) increased the corrosiveness of the salts after 1500 h at 300 °C. These findings suggest that further experimental studies are needed to fully understand the behavior of these nanofluids.

### 3.3. Alumina-Forming Alloys

Alumina-forming alloys, such as FeCrAl (ferritic stainless steels containing 20 wt% Cr and 5 wt% Al), have been extensively studied in recent years due to their ability to generate an Al_2_O_3_ surface layer through heat treatment, yielding promising results to those observed with the incorporation of nanoadditives into salts [72].

Fernández et al. [73] investigated the corrosion resistance of several Al-containing alloys (8-80 wt% Ni, 12-15 wt% Cr, 3-4 wt% Al, 1-3 wt% Nb) in mixtures of nitrates (LiNO_3_-KNO_3_-NaNO_3_, 30-57-13 wt%), carbonates (Li_2_CO_3_-Na_2_CO_3_-K_2_CO_3_, 32-33-35 wt%), and chlorides (LiCl-KCl, 45-55 wt%), at temperatures up to 750 °C for 1000 h under isothermal immersion conditions. The HRR24 (47Ni-21Cr-3Al-27.5Fe wt%) and In702 (75Ni-16Cr-3Al-2Fe wt%) alloys demonstrated the highest corrosion resistance. Furthermore, Fernández proposed another study with two alumina-forming austenitic alloys, OC4 and HR224, exposed to the eutectic ternary salt mixture (Li_2_CO_3_-Na_2_CO_3_-K_2_CO_3_) at 650 °C for 1000 h [23]. The corrosion rates were significantly lower compared to non-alumina-forming austenitic alloys in molten carbonates.

Gómez-Vidal et al. [74] examined the corrosion resistance of In702, HA224, and Kanthal APMT in MgCl_2_-KCl (35.6-64.4 wt%) at 700 °C in an Ar atmosphere for up to 500 h. Pre-oxidation treatments resulted in the formation of alumina layers, which provided passivation by creating protective oxides on the surface. The results indicated that the formation of a dense, uniform alumina layer during pre-oxidation protected the alloys from a molten chloride salt attack, with In702 (the highest Ni content) showing the lowest corrosion rate.

Despite their excellent corrosion resistance in most molten salts, the primary drawback of these alloys is their high cost. Therefore, it is essential to explore cost-effective alumina-forming alloys in order to increase access to high-performance materials for TES systems.

### 3.4. Corrosion Inhibitors

Corrosion inhibitors, such as magnesium and calcium ions, are additives that modify the chemical environment within molten salts, mitigating corrosive reactions by forming protective layers on metal surfaces.

Ding et al. [75] investigated the use of magnesium (1 wt%) as a corrosion inhibitor in chloride salts (MgCl_2_-NaCl-KCl in a 60-20-20 mol% ratio) at 700 °C under an inert atmosphere for 500 h. The results showed a significant reduction in corrosion rates, approximately 83% for SS310, 70% for In800H, and 94% for Hastelloy C-276, compared to tests without magnesium addition. Similarly, Frangini et al. [76] found that the addition of magnesium or calcium at concentrations above 1.5 mol% effectively inhibited the corrosivity of Li_2_CO_3_ -Na_2_CO_3_ molten salt. These findings highlight the effectiveness of alkaline-earth metal additives as corrosion inhibitors in high-temperature molten salt environments. However, the effectiveness of these inhibitors depends strongly on salt composition and operating temperature, implying that their application must be specifically tailored for each TES system.

### 3.5. Coatings

Another option to enhance the corrosion resistance of alloys is to coat steels with protective layers, such as ceramics or metallic coatings, similar to those employed at high temperatures in other systems. The types of coatings used can also be very varied, including nickel-based alloys [77,78], cobalt-based alloys [79,80], or metal matrix composites reinforced with WC, Al_2_O_3_, TiC, or SiC [81,82,83]. The most frequently used oxide is Y_2_O_3_, due to its thermal stability, as well as its ability to reduce particle size and improve the refinement of the host material [84,85]. Another commonly employed oxide is ZrO_2_, which is added to enhance hardness and wear resistance, and also acts as a barrier against corrosion when in a nanocrystalline structure [82,86]. Finally, the addition of ceramic particles in concentrations below 1 wt% has shown beneficial effects at temperatures exceeding 900 °C, thereby expanding the operational range of the steel.

Encinas-Sánchez et al. [87] studied the corrosion resistance of SS340 and P91 in solar salt, with the latter coated using a Sol-gel ZrO_2_-3%mol Y_2_O_3_ layer. Porcayo-Calderon et al. [88] investigated the corrosion resistance of SS304 with Ni20Cr coatings in ZnCl_2_-KCl molten salt. The results indicated that Ni20Cr coatings outperformed uncoated 304 stainless steel, primarily due to the high nickel content of the coating.

Other researchers studied the application of electrodeposition coatings. Kondaiah et al. [89] examined the corrosion behavior of various alloys (SS310, SS316, SS347, and In800H) coated with Ni by electrodeposition in molten salts Li_2_CO_3_-Na_2_CO_3_-K_2_CO_3_ (32-33-35 wt%) at 750 °C. The corrosion rates were reduced by up to 60% compared to the same uncoated stainless steel alloys, and by up to 18% for the high-cost Ni-based alloy HA230. These same authors evaluated the alloys under similar conditions in molten chloride salts MgCl_2_-KCl-NaCl (40-32.5-27.5 wt%) [90]. They found that the coated samples exhibited a considerable reduction in the corrosion rate, between 350 and 480 μm/yr in high-purity salts and between 450 and 490 μm/yr in purified industrial salts. These values are approximately 70 wt% lower than those typically observed in uncoated alloys.

Finally, oxide coatings are ineffective in fluoride-based molten salt environments due to the high reactivity of fluorides, which readily disrupt and degrade oxide layers, making alternative protective strategies necessary for corrosion resistance in such systems.

### 3.6. Graphitization

Finally, an additional corrosion mitigation strategy that has gained attention in recent studies is graphitization. This technique involves the thermal conversion of carbon-containing materials, such as carbon steel, into graphitic structures at the metal–salt interface. This transformation forms a dense, chemically inert graphitic layer that serves to passivate the surface and inhibit corrosion. The layer acts as a diffusion barrier, inhibiting the ingress of aggressive ions, such as chlorides or carbonates, and suppressing oxidation reactions, thereby enhancing material stability under thermal exposure to high-temperature molten salts.

Grosu et al. [91] compared the performance of carbon steel exposed to the hydrated salt HitecXL·H_2_O with and without graphitization. Following cyclic immersion tests conducted in the 300–500 °C range under an air atmosphere, the corrosion rate of graphitized carbon steel was found to be nearly three times lower than that of non-graphitized steel (11.4 ± 1.2 vs. 31.5 ± 1.6 μm/yr). Similarly, Gonzalez et al. [92] investigated spray-graphitization as a corrosion prevention method for molten binary nitrate salts. Their findings revealed that applying graphitization at 390 °C reduced the corrosion rate of carbon steel by approximately 50%. Furthermore, the addition of 2 wt% graphite directly into the salt led to a more than sixfold reduction in corrosion rate compared to untreated carbon steel, attributed to the formation of homogeneously distributed iron carbide crystals on the steel surface. These results underscore the promising potential of graphitization as an effective and innovative anticorrosion technique for thermal energy storage systems.

As discussed in this section on corrosion mitigation strategies, a wide range of potential alternatives is available. Among the most promising are protective coatings in their various forms, surface passivation through the formation of alumina films, and the addition of nanoparticles or corrosion inhibitors such as magnesium and graphite. These approaches have shown considerable potential in enhancing the corrosion resistance of commonly used alloys. As a result, significant progress has been made in improving material performance under harsh operating conditions. However, further experimental research is essential to fully evaluate the effectiveness of each method and to support the development of new, innovative strategies for long-term corrosion protection.

**Table 2:** Review of techniques to mitigate the corrosion phenomenon of molten salts and materials.

Material	Molten Salt	Mitigation Strategies	Conclusion	Ref.
SS310/In800H	MgCl_2_-KCl-NaCl	Molten salt purification	Salt purification decreases corrosion by minimizing the reactive impurities that destabilize oxide layers on metal surfaces.	[67]
SS347	Solar Salt (Refined)	Addition of nanoadditives: Al_2_O_3_, SiO_2_	Solar salt with the addition of 1 wt% Al_2_O_3_ nanoparticle: 0.007 mm/yr Solar salt with the addition of 1 wt% SiO_2_ nanoparticle: 0.022 mm/yr Solar salt without nanoadditives 0.021 mm/yr	[69]
SS347	Solar Salt (Industrial)	Addition of nanoadditives: Al_2_O_3_, SiO_2_	Solar salt with the addition of 1 wt% Al_2_O_3_ nanoparticle: 0.019 mm/yr Solar salt with the addition of 1 wt% SiO_2_ nanoparticle: 0.024 mm/yr Solar salt without nanoparticles: 0.027 mm/yr	
Carbon steel	Solar Salt	Addition of nanoadditives: Al_2_O_3_, SiO_2_	The corrosion layer was approximately three times thinner compared to the fluid without nanoparticles.	[12]
In625	NaCl-KCl-MgCl_2_	Addition of nanoadditives: Al_2_O_3_	Better corrosion behavior compared to the case without additives.	[70]
Carbon steel	NaNO_3_-KNO_3-_Ca(NO_3_)_2_	Addition of nanoadditives: Al_2_O_3_, SiO_2_	Solar salt with the addition of 1 wt% Al_2_O_3_ nanoparticle: 0.013 mm/yr Solar salt with the addition of 1 wt% SiO_2_ nanoparticle: 0.023 mm/yr Solar salt without nanoparticles: 0.0075 mm/yr	[71]
SS304/OC4/OC-T/In702/HR224	LiNO_3_-KNO_3_ -NaNO_3_ Li_2_CO_3_ -Na_2_CO_3_-K_2_CO_3_ LiCl-KCl	Alumina-forming alloys	The corrosion rates were significantly lower compared to non-alumina-forming austenitic alloys	[73]
HR224, OC4	Li_2_CO_3_-Na_2_CO_3_-K_2_CO_3_	Alumina-forming alloys	Both alloys present corrosion rates lower than those reported for similar alloys in molten carbonates.	[23]
In702, HR224, Kanthal APMT	MgCl_2_-KCl	Alumina-forming alloys	Pre-oxidation was used to form protective oxides, surface passivation.	[74]
SS310, In800H, C276	MgCl_2_-KCl-NaCl	Corrosion inhibitor: Mg	The corrosion rates were found to be significantly reduced by about 83% for SS310, 70% for In800H, and 94% for C276 compared with the exposure tests without Mg addition.	[75]
SS316L	Li_2_CO_3_-Na_2_CO_3_	Corrosion inhibitor: Mg, Ca	As the concentration of Mg and Ca additives increased (over 1.5% mol) further, the SS316L became more corrosion resistant.	[76]
P91	Solar Salt	Coating: ZrO_2_-Y_2_O_3_	The results clearly show the good behavior of the coated samples.	[87]
SS304	ZnCl_2_-KCl	Coating: Ni20Cr	The corrosion results described clearly that a high Ni content of the alloy is very effective in improving the corrosion resistance while Cr plays a detrimental role.	[88]
S310, SS316, SS347, In800H	Li_2_CO_3_-Na_2_CO_3_-K_2_CO_3_	Coating: fractal-textured Ni electrodeposition	The corrosion rate of double-layer Ni coatings on ferrous alloys was reduced by as much as 60% from that of uncoated surfaces.	[89]
SS310, SS316, SS347, In800H	MgCl_2_-KCl-NaCl	Coating: fractal-textured Ni electrodeposition	The results are 70% lower than those typically observed in uncoated alloys.	[90]
Carbon steel	HitecXL·H_2_O	Graphitization	Corrosion rate of graphitized carbon steel: 11.4 ± 1.2 µm/yr Corrosion rate of non-graphitized carbon steel: 31.5 ± 1.6 µm/yr	[91]
SS310, SS347	Li_2_CO_3_-Na_2_CO_3_-K_2_CO_3_	Graphitization	A considerable improvement in corrosivity was achieved when graphitization was applied.	[45]
Carbon steel	Solar Salt	Graphitization	Corrosion rates were reduced almost twice when graphitization was applied.	[92]

## 4. Discussion

This review offers a comprehensive overview of corrosion phenomena and mitigation strategies in molten salt-based thermal energy storage (TES) systems, emphasizing the importance of managing corrosion in high-temperature environments. Key findings reveal that corrosion rates are significantly affected by factors such as salt composition, the presence of impurities, alloy type, operating temperature, and flow conditions. These insights underscore the need for integrated and system-specific strategies to effectively address corrosion challenges in TES applications. The principal findings of this study are summarized below.

1. Evaluating the compatibility between the storage medium and structural materials, along with the specific operating conditions, is critical for the reliable performance of TES systems. Different materials exhibit varying degrees of compatibility with specific molten salts, highlighting the importance of careful material selection. Alloys with high chromium and nickel content tend to offer better corrosion resistance in more aggressive environments, such as chloride and carbonate salts. In contrast, stainless steels and carbon steels have shown effective performance in nitrate-based salts, particularly at moderate operating temperatures. This compatibility assessment is key to ensuring long-term durability and efficiency in TES applications.
2. One of the most significant contributions of this review is its analysis of dynamic corrosion, a topic often overlooked in favour of simpler static testing. Although dynamic conditions more accurately reflect real-world TES environments, most previous studies have focused on static setups because of their simplicity. This review emphasizes how factors such as flow rate and thermal cycling accelerate corrosion by removing protective oxide layers and exposing fresh metal surfaces to corrosive molten salts. These erosion–corrosion mechanisms play a critical role in long-term material degradation, highlighting the importance of considering dynamic corrosion when evaluating TES materials. By addressing this gap, the review also underscores the need to investigate real plant conditions, such as those in electric heaters, where temperature cycling and the formation of hot spots further complicate corrosion behavior and require deeper exploration.
3. The purification of molten salts to eliminate reactive impurities, especially chlorides, or the use of high-purity salts, plays a crucial role in influencing corrosion mechanisms. In parallel, the addition of corrosion inhibitors, such as magnesium (Mg) and calcium (Ca), presents an effective alternative for significantly reducing corrosion rates. These approaches are not only practical and cost-effective but are also highly relevant for extending the service life of components used in thermal energy storage (TES) systems. By minimizing corrosive interactions, these strategies contribute to the long-term reliability and economic feasibility of TES technologies.
4. Various additional strategies have been explored to enhance corrosion resistance in TES systems, including the use of additives, alumina-forming alloys, and protective coatings. Incorporating alumina and silica nanoparticles into molten salts has shown promising potential in improving corrosion resistance by stabilizing protective layers on material surfaces. Alumina-forming alloys demonstrate strong resistance in aggressive salt environments, especially in chloride and carbonate-based salts. However, to make these high-performance materials more widely accessible, the development of cost-effective alumina-forming alloys is essential for the broader implementation of TES technologies. The alternative of protective coatings has also been analyzed; however, they present certain limitations, such as their applicability to fluoride-based salts and the requirement for 100% coverage to be effective.

Despite significant advances in understanding corrosion in molten salt-based TES systems, several critical challenges remain. Experimental data are still limited for many combinations of molten salts and structural alloys, making it difficult to generalize compatibility trends. Most available studies have focused on a narrow range of salt compositions and operating conditions, leaving gaps, particularly at higher operating temperatures, which are increasingly relevant for next-generation systems. Furthermore, most experimental work has been conducted under static conditions that fail to fully replicate the complex, dynamic environments found in real-world TES applications. Specific power-to-heat technologies, such as electric heaters, remain insufficiently characterized in terms of corrosion behavior. In particular, hot spots and elevated film temperatures on heating surfaces can significantly accelerate localized corrosion and degrade materials. These thermal gradients and their associated effects must be carefully evaluated to ensure the safe and reliable operation of such components in molten salt environments.

To address these limitations, future research should prioritize a more comprehensive investigation of corrosion mitigation strategies, particularly under dynamic conditions that reflect operational realities. The development of advanced experimental setups that allow for the simulation of flow and thermal cycling will be essential to gain deeper insights into long-term material performance. These efforts aim to bridge the gap between laboratory-scale studies and real plant conditions, ultimately enhancing the long-term viability of TES infrastructure.

## 5. Conclusions

The current energy landscape highlights the crucial role of energy storage systems, emphasizing the importance of analyzing the phenomenon of corrosion between molten salts and contact material. This review synthesizes the most relevant studies in literature, leading to the following conclusions.

- Although the corrosion behavior of alloys in molten salts has been extensively studied under static conditions, this review highlights that corrosion rates generally increase under flow conditions, even when experimental parameters remain constant. Therefore, it is essential to prioritize corrosion studies under dynamic conditions that better reflect real plant operations.
- Corrosion behavior is influenced by several interdependent factors, including temperature, impurities, the chemical composition of alloys and molten salts, the surrounding gas atmosphere, and flow rate. A comprehensive understanding of these variables is crucial for developing effective mitigation strategies.
- Various corrosion mitigation approaches, such as the addition of nanoparticles, the use of corrosion inhibitors, and the application of protective coatings, have shown promise. However, these strategies require further optimization and validation under realistic operational conditions.

In conclusion, despite extensive research on corrosion phenomena, significant knowledge gaps remain. This review serves as a foundational reference for addressing these gaps, highlighting the need for ongoing research to better understand and mitigate corrosion in energy storage systems. Therefore, this research contributes to the development of durable and efficient molten salt-based thermal systems, supporting their long-term viability for renewable energy and industrial decarbonization.

## Data Availability

No new data were created or analyzed in this study. Data sharing is not applicable to this article.
